# Evolutionary toxicology: Toward a unified understanding of life's response to toxic chemicals

**DOI:** 10.1111/eva.12519

**Published:** 2017-11-10

**Authors:** Steven P. Brady, Emily Monosson, Cole W. Matson, John W. Bickham

**Affiliations:** ^1^ Biology Department Southern Connecticut State University New Haven CT USA; ^2^ The Ronin Institute for Independent Scholars and Department of Environmental Conservation University of Massachusetts Amherst MA USA; ^3^ Department of Environmental Science and Center for Reservoir and Aquatic Systems Research (CRASR) Baylor University Waco TX USA; ^4^ Department of Wildlife & Fisheries Sciences Texas A&M University College Station College Station TX USA

Darwin himself could scarcely have found a better example of the operation of natural selection than is provided by the way the mechanism of resistance operates.

Carson, 2002

Over the course of billions of years, receptors and the organisms in which they function have been evolving endocrine systems that are astonishing in their complexity, diversity, and biological importance. Protecting the life forms and ecosystems that have emerged from this evolutionary process will require that our policies take account of these characteristics.

Thornton, 2003

## 1 A HISTORICAL PERSPECTIVE ON EVOLUTIONARY TOXICOLOGY

The story of life on Earth is one of both ancient and ongoing evolution. All species on the planet today have in different ways evolved adaptations that promote fitness sufficiently well enough to sustain different meta‐populations over long periods of time relative to the pace of environmental change. Indeed, species’ lifespans are estimated to be on the order of millions of years (Barnosky et al., 2011). At the same time, we now appreciate that evolution is a contemporary process that modifies traits and shapes fitness each and every generation (Carroll, Hendry, Reznick, & Fox, 2007; Hendry & Kinnison, 1999). As we show in this special issue, these two elements of evolutionary change—macroevolutionary diversification and contemporary evolutionary change—bear critical insights for ecotoxicology and point toward a fruitful integration of the fields of toxicology and evolutionary biology.

Many adaptations that have arisen over macroevolutionary timescales reflect responses to selection imposed by toxins that characterized the early environment on the planet (Kirschvink & Kopp, 2008; Monosson, 2012; Tobler et al., 2011). Indeed, much of the early evolution of life, from its origins to the evolution of multicellular plants and animals, faced a central problem of evolving mechanisms for coping with toxicity imposed, for example, by heavy metals, ultraviolet light, oxygen, microbial toxins, and defensive chemicals produced by plants (Cockell, 1998; Coyle, Philcox, Carey, & Rofe, 2002; Kirschvink & Kopp, 2008; Rico, 2001). For modern ecotoxicology, evolutionary history suggests that in some cases, extant species may already possess pre‐adaptations or adaptive capacity for dealing with exposure to toxicants (sensu Motychak, Brodie, & Edmund, 1999; Llewelyn et al., 2011). Further, closely related species may share similar tolerances to similar toxicants (Guénard, von der Ohe, de Zwart, Legendre, & Lek, 2011). Therefore, the evolutionary history of a given species or group of species may provide an important source of variation associated with tolerance to contaminants found in the environment today (e.g., Hammond, Jones, Stephens, & Relyea, 2012). This predictive capacity may be particularly true for historical toxins that have been re‐mobilized as contaminants by recent human activities (e.g., via land use practices, agriculture, and mining). In contrast, adapting to novel contaminants such as synthetically produced chemicals with no precedent of occurrence in the environment may prove especially challenging, for example, if adaptive responses require novel genetic variation (Barrett and Schluter 2008). Moreover, the occurrence of contaminants alongside numerous other human‐induced selection pressures (e.g., climate change, ocean acidification, habitat conversion, commercial harvest) may further challenge the ability of organisms to adapt to environmental contaminants. After all, an individual's fitness is influenced by the sum total of all stressors, which can act additively, antagonistically, and/or synergistically.

In addition to the influence that macroevolution has had on species’ tolerance for toxins and toxicants, ongoing contemporary evolutionary change mediates tolerance over time periods relevant to policy and conservation. Although this view of evolution as a contemporary process has only recently become more prevalent in toxicology (Bickham, 2011), the awareness of the potential for organisms to adapt to environmental toxicants dates back to at least the early 20th century. At that time, Melander (1914) reported an experiment showing a reduced effectiveness of sulfur‐lime treatments on the agricultural pest *Quadraspidiotus perniciosus* (San Jose scale), a result contrasting the usual effect of complete mortality. This example appears to be the first reported evidence of pesticide resistance. Three decades later in 1945, with use of the miracle drug penicillin on the rise, Alexander Fleming saw fit to conclude his Nobel Lecture with a cautionary tale about the possibility of the evolution of antibiotic resistance, forewarning the inefficacy of treatment that would follow (Nobelprize.org). These early examples of resistance provided some of the first evidence that evolution can be quite rapid and that evolutionary change can have important ramifications for human health and the economy (Palumbi, 2001).

By the middle of the 20th century, more formal evidence for evolutionary responses to a diversity of toxicants began to mount, with reports of pesticide resistance on the rise. Indeed, in her renowned book, Rachel Carson devoted an entire chapter of Silent Spring to the evolution of pesticide resistance (2002). Around that time, in addition to numerous reports of invertebrates and plants evolving pesticide and industrial contaminant resistance (e.g., Antonovics, Bradshaw, & Turner, 1971; Keiding & Van Deurs, 1949; March & Metcalf, 1949), some of the first examples of toxicant resistance in vertebrates were beginning to emerge. While artificial selection experiments demonstrated the capacity for increased tolerance to DDT in mice (Ozburn & Morrison, 1962), studies of wild populations indicated that resistance was evolving in nature. For instance, Boyle (1960) showed that a rat population on a farm in Scotland had evolved resistance to two different poisons (warfarin and diphacinone). Around the same time, multiple populations of frogs and fish had evolved resistance to DDT and other insecticides applied to farm fields (Boyd & Ferguson, 1964; Boyd, Vinson, & Ferguson, 1963).

As these examples of resistance grew, it was beginning to look like our industrial age chemicals and cures were contributing to a new phenomenon: rapid evolution in response to toxic chemicals mobilized by human activities. Yet for much of the twentieth century, these early examples of resistance to pesticides and antibiotics had been set aside from mainstream evolutionary biology, considered instead to be special cases of evolution acting unusually rapidly. Recently, however, this view of evolution has changed. It is now clear that these early accounts of resistance were not exceptional cases of fast evolution. Rather, these examples were just the beginning of what is sometimes referred to as the “newest evolutionary synthesis,” reflecting our recent understanding that contemporary evolutionary changes—those occurring in just a few generations—are common and widespread (Hendry, Gotanda, & Svensson, 2017; Schoener, 2011). While such changes have been detected in natural contexts such as in response to variation in food resources (Grant & Grant, 2002) or predation pressures (Reznick, Shaw, Rodd, & Shaw, 1997), many recent examples have emerged in human‐modified contexts (Carroll et al., 2014; Hendry et al., 2017; Smith & Bernatchez, 2008), including climate change (Norberg, Urban, Vellend, Klausmeier, & Loeuille, 2012), land conversion (Alberti et al., 2017; Brady & Richardson, 2017), invasive species (Novak, 2007), and commercial harvest (Heino, Díaz Pauli, & Dieckmann, 2015).

Recently, we have also developed a greater awareness of a role for toxic chemicals in contemporary evolution, including the capacity of toxins and toxicants to mediate genetic change, plasticity, and epigenetic effects. As a result, our view of toxic chemicals has shifted, such that we are no longer limited to assessing chemicals exclusively for their acute or chronic intragenerational toxic effects. Instead, we increasingly see the potential for toxic chemicals to cause transgenerational effects mediated, for example, by natural selection. Undoubtedly, the emergence of industrial era chemicals has created—and continues to create—profoundly different selective environments for organisms on the planet today. Indeed, there now exists an amazing diversity of industrial chemicals. For instance, the Toxic Substance Control Act Chemical Substance Inventory lists over 85,000 chemicals on the U.S. market (U.S. Government Accountability Office 2013). Coupled with the extraction and global redistribution of chemical and radioactive elements, minerals, and compounds once found primarily within Earth's crust (e.g., mercury, lead, cadmium, uranium, hydrocarbons), the combined novelty, intensity, and scope of modern day contaminants are, as best we can tell, unprecedented.

The extent to which evolution will contribute to the success of populations facing this new suite of pressures remains uncertain. Attempting to understand this capacity of evolution is a recurrent theme in the papers appearing in this special issue (see especially Whitehead et al. 2017). Our ability to gain this understanding will surely improve as we broaden our assessment of evolutionary toxicology beyond the scope of pesticide resistance, and focus on increasingly diverse contexts where nontarget organisms are impacted by the use and distribution of toxic chemicals in the environment (e.g., Hua et al. 2017; Whitehead, Clark, Reid, Hahn, & Nacci, et al. 2017). Indeed, numerous observations of phenotypic and molecular changes have now been described in wild populations exposed to industrial age chemicals. These changes are diverse, ranging from the evolution of phenological traits to desensitization of aryl‐hydrocarbon receptors to increased DNA mutation rates to epigenetic effects (Bélanger‐Deschênes, Couture, Campbell, & Bernatchez, 2013; Bickham, 2011; Bickham, Sandhu, Hebert, Chikhi, & Athwal, 2000; Crews & Gore, 2012; Kiang, 1982; Oziolor, Bigorgne, Aguilar, Usenko, & Matson, 2014; Oziolor & Matson, 2015; Reid et al., 2016; Yauk, Fox, McCarry, & Quinn, 2000). Notably, however, not all changes are adaptive. Rather, it appears that in some cases, populations can evolve maladaptive responses to contaminants (e.g., Brady, 2013; Rogalski, 2017; Rolshausen et al., 2015). For example, strong selection pressures such as those from contaminants can reduce population size and lead to inbreeding depression or drift (sensu Falk, Parent, Agashe, & Bolnick, 2012). Moreover, contaminant‐induced selection can reduce genetic variation, which can limit capacity for adaptive responses to future stressors.

Together, these various biological changes induced by toxic chemicals highlight the complexity of outcomes that we can begin to understand when evolution is considered in the context of ecotoxicology. Solidifying the field of evolutionary toxicology should help resolve these complexities as we continue to elucidate the relationships between toxic exposure, genetic architecture, molecular pathways, trait variation, and population responses. Indeed, it is our hope that this special issue will not only showcase the recent advances in ecotoxicology availed by evolutionary perspectives but also catalyze a more unified field of study that routinely considers the role of evolution in governing life's responses to toxic chemicals.

## 2 EVOLUTIONARY TOXICOLOGY—BUILDING A FOUNDATION FOR TOXICOLOGY

Having emerged in response to the need for human health and environmental regulatory policies, toxicology as a field has been the workhorse science of numerous industries and regulatory agencies (Monosson, 2005). In fact, one hypothesis concerning the field's development is that the “discipline expands in response to legislation” (Gallo & Doull, 1996). And yet there currently exists little if any consideration of evolution in policymaking or regulatory processes relating to environmental contaminants. However, as reports of resistance to antibiotics, insecticides, herbicides, and other industrial age chemicals rise, the relevance of evolutionary responses to toxic chemicals should become increasingly apparent. For instance, failing to consider the influence of evolutionary responses to toxicants can result not only in quantitative error but also qualitatively different inferences (Brady & Richardson, 2017; Oziolor, De Schamphelaere, & Matson, 2016). As well, by failing to consider the evolutionary history of biological systems involved in defending life from natural toxins (e.g., Goldstone et al. 2006), we may miss opportunities to predict how life might respond to industrial age chemicals that interact with these systems.

As interest in evolutionary toxicology begins to rise (Coutellec & Barata, 2011, 2013), the time is ripe to examine the breadth of this foundational perspective. Elements of evolutionary biology have started to become utilized in various aspects of toxicology. For example, theoretical and analytical studies have developed and incorporated evolutionary techniques for ecotoxicology (e.g., Bélanger‐Deschênes et al., 2013; Klerks, Xie, & Levinton, 2011) while conceptual studies have developed frameworks for integrating evolution and ecotoxicology (e.g., Leung et al., 2017). Empirical studies have increasingly been detecting evolutionary responses in diverse environmental contexts, for example, in response to mining effluents and industrial pollutants (Bougas et al., 2016; Chen et al., 2015; García‐Balboa et al., 2013; Laporte et al., 2016; Reid et al., 2016). Studies are also beginning to show that adaptation to toxicants can evolve at a cost, for example, in the form of increased sensitivity to oxidative stress following adaptation to PCBs (Harbeitner, Hahn, & Timme‐Laragy, 2013). As a result, we are beginning to appreciate that potential consequences of adaptation should be carefully considered when evaluating the potential for evolution to mediate current and subsequent environmental impacts.

Despite these recent efforts to draw upon and promote evolutionary perspectives in ecotoxicology, the various insights and ideas found in the literature remain fragmented. That is, although there have been various efforts at unifying evolutionary and ecotoxicological approaches toward a more holistic understanding of toxicity, ecotoxicology still largely lacks an evolutionary perspective. Indeed, it was our perception of this disconnect that motivated us to compile this special issue. As we began searching for potential contributions, we found relatively few researchers actively integrating toxicology and evolution. Our goal with this special issue is to bring together and showcase these diverse perspectives, approaches, and insights under one broad umbrella of *evolutionary toxicology*. In doing so, we hope to cultivate a more fruitful pursuit toward the understanding and mitigation of the negative biological and ecological impacts of chemical contaminants. As with other applied fields strengthened with evolutionary perspectives (e.g., medicine, public health, agriculture, conservation biology), we feel that embracing a heightened awareness of the influence of evolutionary processes on life's response to toxic chemicals will provide ecotoxicologists with a deeper understanding that improves capacity for predicting the consequences of contaminants on populations, communities, and ecosystems.

At the same time, the infusion of evolutionary principles into the traditionally applied field of toxicology should provide reciprocal benefit to evolutionary biology. Specifically, evolutionary investigations in toxicology should lead not only to more insightful assessments of contaminant exposure risk but should also expand the range of systems in which to study evolutionary processes and outcomes (Monosson, 2012) and in particular advance the growing interest in understanding evolution in human‐modified environments (Alberti et al., 2017; Hendry et al., 2017). Moreover, with rapid advances of methods in genomics, transcriptomics, and other fields, it is likely that evolutionary toxicology will expand mutually beneficial insights far beyond what we can envision today (Oziolor, Bickham, & Matson, 2017).

## 3 IN THE ISSUE

In this special issue**,** Whitehead et al. (2017) synthesize insights from a well‐studied example of contemporary evolution, in which wild populations of *Fundulus heteroclitus* (Atlantic killifish) have adapted to some of the most polluted estuaries in the U.S.A. The authors review how attributes of populations, their genetic architecture, and characteristics of contaminants may be key in determining the likelihood for adaptive responses substantial enough to promote population persistence. The authors suggest that extremely high genetic variation may have been a distinguishing feature facilitating a successful adaptive response among *F. heteroclitus* populations in highly contaminated sites. Critically, the authors note that evolution alone may only rarely be a fast enough “solution to pollution.” In another example of contemporary evolution in a contaminated marine environment, Lee et al. (2017) report the near real‐time adaptation of populations of the common copepod *Eurytemora affinis* in response to the 2010 BP *Deepwater Horizon* oil spill. Cited as the most catastrophic oil spill in U.S. history, this event is estimated to have leaked 4.6 million barrels of oil into the Gulf of Mexico (Griffiths 2012). The experimental design used by Lee et al. (2017) is quite rare, in that the authors studied populations collected before and after the oil spill. Populations collected after the oil spill showed evidence of adaptive evolution, indicated by rapid development and an increased tolerance to crude oil, relative to populations collected prior to the oil spill. However, a laboratory‐based selection experiment conducted for eight generations (5–6 months) failed to fully replicate the field results, showing the evolution of development time, but not of tolerance to crude oil. These results revealed that rapid evolution of tolerance could take place in response to the toxic effects of crude oil, but perhaps require more time or more genetic variation than was present in the laboratory selection experiment.

Epigenetic mechanisms are increasingly considered as candidates mediating transgenerational responses to environmental stressors. Like Lee et al. (2017), Robertson, Schrey, Shayter, Moss, and Richards (2017) also took the opportunity presented by the *Deepwater Horizon* oil spill of 2010 to investigate the potential roles of both genetic and epigenetic changes in *Spartina alterniflora* (a common saltmarsh grass). *Spartina alterniflora* has earned a reputation as notably resilient to various forms of severe environmental disturbances, and this species, like the ubiquitous killifish, has been studied for phenotypic and genetic differences across a range of coastal habitats. Examining the genetic and epigenetic characteristics of recovering populations of *S. alterniflora,* the authors report genetic differentiation in response to oil exposure, but were unable to detect epigenetic differentiation between oil exposed and unexposed populations. Their contribution provides insight into the rapidly expanding field of epigenetics, particularly as it may relate to transgenerational responses to environmental disruptions. In complement, Oziolor et al. (2017) discuss how omics approaches provide tools to expand the assessment of populations under chemical duress, particularly when contamination leads to multigenerational, population‐wide impacts. Their work is framed as an update to the “four cornerstones of evolutionary toxicology” described by Bickham (2011), with an emphasis on new areas of study opened up by rapidly advancing omics technologies and tools.

Dutilleul et al. (2017) observed that two different contaminants can drive opposite adaptive evolutionary strategies in a single species. Using an experimental evolution approach, the authors show that *Caenorhabditis elegans* can evolve faster life histories in response to uranium contamination but slower life histories in response to sodium chloride. Surprisingly, they also found that populations exposed to both toxicants in alternation evolved similar or higher fitness compared to populations evolving in response to either of the toxicants in singularity. Moreover, evolution in response to this alternating toxicant regime did not reduce fitness in the original (uncontaminated) environment. Thus, adaptation to alternating toxicants did not induce a fitness cost to a former environment. This work further highlights the complexity of toxicological responses and prompts future consideration of the processes that may limit fitness in the contexts of contaminated environments with constant versus heterogeneous exposure regimes. Notably, they report on two stressors—uranium and sodium chloride—that are increasing in the environment as a result of human activities.

In Hua et al. (2017), recently evolved pesticide tolerance in an amphibian is shown to influence its susceptibility to parasites. Notably, adaptation to pesticides can in some cases incur collateral adaptive benefits. Here, the authors show that adaptation to a pesticide is linked with resistance to a common trematode parasite. However, the authors also show that adaptation to a pesticide can bear costs, as indicated by increased viral loads following exposure to ranavirus. This work from Hua et al. (2017) highlights the need to consider multiple stressors in evolutionary ecotoxicological inquiry and reminds us of the value of studying nontarget species in agricultural contexts, where most of our knowledge about evolution has been limited largely to studies of pest species. Another important takeaway from Hua et al. (2017) is that community context matters when assessing the evolutionary impacts of contaminants on a given organism, a point seldom considered in community ecotoxicology. Thus, Hua et al. (2017) bring a much needed community perspective to evolutionary toxicology.

This special issue also provides insights into how the application of evolutionary principles in ecotoxicology can strengthen both predictions and conclusions. Brady, Richardson, and Kunz (2017) discuss the evolutionary history of responses to osmotic stressors, focusing on the toxicity of chloride (both an ancient toxin and a modern toxicant) and the potential capacity of freshwater organisms to adapt to salt pollution. By demonstrating a phylogenetic signal of chloride tolerance across 55 freshwater species, the authors highlight the potential utility of evolutionary relationships in predicting untested species’ responses to chloride exposure. Brady, Richardson, and Kunz (2017) also point out that contemporary evolution may result in differences in contaminant tolerance among local populations, with the magnitude of differences between populations matching that seen between species. Given that these differences are likely to increase over time, contemporary evolutionary perspectives may alter our perception of the protectiveness of water quality criteria for freshwater species.

Like salt, ionizing radiation is a toxin and toxicant as old as the planet. While defensive responses to ionizing and nonionizing radiation—such as DNA repair and antioxidant capacity—likely appeared at the dawn of life and have been conserved (in most species) for over 3 billion years, humanity has recently upped the ante with nuclear bombs and nuclear power plants. Baker et al. (2017) explore the consequences of radiation exposure in wild rodents. Based on a study employing full mitochondrial genome sequences, they found increased genetic diversity in native rodent populations inhabiting contaminated environments near the damaged Chernobyl reactor. They conclude that an increase in the mitochondrial mutation rate was plausibly caused by multigenerational, continuous low‐dose radiation exposure in contaminated environments. This finding suggests that toxicants can not only lead to reduced genetic diversity by acting as agents of natural selection but can also accelerate mutation rates and thus increase the possibility for novel adaptive and maladaptive genetic variation (e.g., genetic or mutational load).

With roots set into the ground, terrestrial plants have been easy targets for predators. This predation pressure may explain in part the great diversity of phytochemicals exhibiting varying degrees of toxicity. A recent and growing perspective on phytochemicals considers how some chemicals share properties with vertebrate hormones thus having the potential to interact with vertebrate endocrine systems. Recent evidence suggests that exposure of vertebrates to such “hormonally active phytochemicals” can influence behavior, physiology, and fitness. Here, Lambert and Edwards (2017) explore how exposure of vertebrates to phytochemicals may present not only selection pressures, but also opportunities for vertebrates to co‐opt benefits from ingesting these chemicals. This work highlights a seldom‐considered view of how ecological and behavioral interactions can mediate the toxicity of a given contaminant.

## 4 FUTURE DIRECTIONS

The articles in this special issue provide both an overview and a current snapshot of evolutionary understanding and application in ecotoxicology. We hope this collection will draw wide attention to and accelerate the incorporation of evolutionary approaches in ecotoxicology, raising awareness of the complementary efforts of these two fields in hopes that they will serve to strengthen each other. For example, looking through an evolutionary lens, ecotoxicologists might consider the impact of industrial chemicals on the development of the toxic response in individuals and in populations. Are there examples of comparable periods in the evolutionary record (e.g., natural yet sudden shifts in the chemical environment), and can we trace the evolution of tolerances to different contaminants through comparative phylogenetic approaches? Can we draw on our current and future understanding of evolution to help identify genetic and physiological mechanisms that are particularly sensitive, resistant, or responsive to classes of contaminants? A focus on evolutionary history may also provide insights into the networked nature of life's response to toxicants. Would this in turn allow us to make better use of omics and molecular pathway approaches in toxicology? All of which may be useful as green chemists work to design chemicals that theoretically pose fewer adverse effects. And as ecotoxicologists and managers are now faced with interpreting and communicating this unprecedented volume of data, would a deeper, evolutionary‐based understanding of the nature of these systems help us glean synthetic insights, identify responsive nodes, or distinguish subtle yet key interspecific differences in responsiveness? Likewise, what of other populations that like various species of fish have managed to survive highly toxic environments (e.g., Wirgin et al., 2011; Bélanger‐Deschênes et al., 2013; Whitehead et al. 2017)? Are these examples the rule or exception, or something in between?

Ecotoxicology is ripe for a revolution. Embracing both evolutionary principles and the evolutionary histories of life's defensive systems and networks promises to transform our capacity to understand and manage the biological impacts of toxic chemicals. The understanding that has been achieved in evolutionary biology and ecotoxicology offers critical knowledge for both fields. Indeed, a greater interaction between these disciplines will lead to a more holistic understanding of the ways that organisms respond to contaminants in the environment. Evolutionary toxicology exemplifies how the application of evolutionary principles can benefit applied toxicology and ecotoxicology. A world of understanding awaits. We hope that this special issue will spark a new generation of inquiries, motivated by the profound influence that evolution has had and can have on the ways that organisms respond to and cope with natural and human‐mediated environmental contaminants.
